# Development of a model of HTLV-1 oral transmission in the rabbit

**DOI:** 10.1186/1742-4690-8-S1-A19

**Published:** 2011-06-06

**Authors:** Robyn Haines, Rebeccah Urbiztondo, Rashade Haynes, Stefan Niewiesk, Michael Lairmore

**Affiliations:** 1 Department of Veterinary Biosciences, The Ohio State University, Columbus, Ohio, 43210, USA; 2 Department of Veterinary Biosciences, Center for Retrovirus Research, The Ohio State University, Columbus, Ohio, 43210, USA; 3 Comprehensive Cancer Center, Arthur G. James Cancer Hospital and Solove Research Institute, The Ohio State University, Columbus, Ohio, 43210, USA

## 

A primary route of transmission of human T-lymphotropic virus 1 (HTLV-1) is from mother-to-child via breast milk, but knowledge of the early immunologic events in orally acquired HTLV-1 infection is limited. Herein, we characterized normal rabbit gut-associated lymphoid tissues (GALT) and performed studies to develop an oral model of HTLV-1 infection. Mononuclear leukocytes were immunophenotyped from key GALT inductive and effector sites using flow cytometry and immunohistochemistry. Our data indicate that unexposed rabbits GALT have a predominant CD4+ lymphocyte population similar to humans. To establish a HTLV-1 oral model 12 week old female New Zealand White rabbits were orally or intravenously inoculated with CD3+CD4+CD25+ rabbit lymphocyte cell line immortalized with the HTLV-1 molecular clone ACH (R-49 cells) or control Jurkat T-cells orally. The rabbits were monitored for hematologic and virologic parameters prior to serial sacrifice. Collectively, 66 to 100% of HTLV-1 orally exposed rabbits became persistently infected. HTLV-1orally exposed and infected rabbits during early time points (1-4 weeks post exposure) had delayed and often less intense anti-HTLV-1 antibody response, variable leukocytosis, and a delayed p19 matrix antigen production and proviral DNA amounts in peripheral blood leukocytes compared to IV exposed rabbits. Interestingly, by 8 weeks post exposure orally exposed rabbits had established similar systemic spread of the virus compared to IV exposed rabbits. This oral model of HTLV-1 transmission in rabbits creates to opportunity to test the role of the mucosal microenvironment during the early stages of orally-acquired HTLV-1 in gut-associated lymphoid tissue.

